# Overlapping cytokines in *H. pylori* infection and gastric cancer: A tandem meta-analysis

**DOI:** 10.3389/fimmu.2023.1125658

**Published:** 2023-03-15

**Authors:** Bingting Yu, Luochengling Xiang, Maikel P. Peppelenbosch, Gwenny M. Fuhler

**Affiliations:** Department of Gastroenterology and Hepatology, Erasmus University Medical Center, Rotterdam, Netherlands

**Keywords:** gastric cancer, *H. pylori*, interleukin-6, cytokines, serum, ELISA

## Abstract

**Background:**

Previous evidence indicated that *Helicobacter pylori*-induced inflammation is the first step towards gastric carcinogenesis. However, investigations of the immunological factors driving this process have shown inconsistencies. We aimed to present a thorough summary of all researched cytokines in relation to *H. pylori* infection and GC and relate these to global GC risk.

**Methods:**

We performed a systematic review and tandem meta-analysis identifying all published studies reporting on serum cytokine levels in *H. pylori*-infected cases vs. non-infected controls and gastric cancer cases vs. non-gastric cancer controls, with sub-analyses performed to identify global regional differences in cytokine induction and their correlation with GC incidence.

**Results:**

Only levels of systemic IL-6 (standardized mean difference [SMD]:0.95, 95%CI [0.45;1.45]) and TNF-α (SMD:0.88, 95%CI [0.46; 1.29]) were significantly increased upon *H. pylori* infection. Sub-analysis showed that of IL-6 levels were increased upon *H. pylori* infection in East Asian, Middle Eastern and Southeast Asian cohorts, but not in North America, Europe, Russia and Africa. Serum levels of IL-6, IL-7, IL-10, IL-12, and TNF-α were significantly raised in GC. Exploration of the relationship between serum cytokines changes upon *H. pylori* infection and regional differences in risk of GC development indicated that the SMD of IL-6 serum levels presents a significant correlation with the relative incidence of GC (*r*=0.81, *p*=0.00014).

**Conclusion:**

This study shows that *H. pylori* infection and GC are associated with increased IL-6 and TNF-α levels. Particularly, IL-6 shows region-specific increases that correlate with GC incidence, making it a key contender for the cause of this disease.

## 1 Introduction

According to the International Agency for Research on Cancer (IARC) of the World Health Organization, gastric cancer (GC) is one of the most deadly cancers worldwide. In the year 2020, over one million patients were diagnosed with GC, and 768,793 people died from this disease globally (Cancer today [https://gco.iarc.fr/today]). The development of GC is a multi-staged and convoluted process. The largest risk factor for GC development is infection with the Gram-negative, microaerophilic, spiral shaped bacterium *Helicobacter pylori* (*H. pylori*) (1). Research has shown that *H. pylori*–induced gastritis is the first step towards gastric carcinogenesis, which is followed by gastric intestinal metaplasia, dysplasia, and cancer (2). However, the exact molecular mechanisms underlying gastric carcinogenesis remain largely unknown.

Evidence that confirms the connection between gastritis and *H. pylori* has started to emerge since the first recognition of this bacterium as potential pathogen by Warren and Marshall in 1983 (3). A natural *H. pylori* infection can last a lifetime, despite inducing an immune response. As such, *H. pylori*-induced stimulation of inflammatory signaling in epithelial and immune cells can cause an accumulation of cytokines and chemokines during the chronic inflammatory phase (4). Cytokines such as IL-1, IL-4, IL-6, IL-10, IL-11, IL-12, TNFα were reported to be raised. Several of these factors serve as trophic agents for epithelial cells, and may stimulate gastric epithelial proliferation (5, 6). In addition, cytokine-induced production of reactive oxygen species can contribute to DNA damage, inducing progression of gastritis to metaplasia, dysplasia and/or cancer (7). Moreover, inflammatory cytokines such as IL-1 and IL-6 can induce the production of angiogenic factors like VEGF (8), while TNFα has the potential to promotes cancer cell proliferation, invasion, and metastasis, as well as tumor angiogenesis (9). Nevertheless, despite the fact that many of these cytokines have been the subject of extensive research, it is not known exactly how specific cytokines individually contribute to the process of gastric carcinogenesis, and findings are sometimes inconclusive (10, 11). While *in vitro* studies have shown the capacity of *H. pylori* to induce diverse cytokines, *ex vivo* measurements of cytokines in serum from patients has not always yielded comparable results (12–15). As *H. pylori* infection rates as well as GC incidence varies globally, differences in regional cohorts may account for some of the variability observed. Therefore, we hypothesized that the immune response would vary depending on the global location the cohorts included or strains of *H. pylori* studied. Knowing which cytokines are induced in response to *H. pylori* infection in which global regions may advance our understanding of the mechanisms behind gastric carcinogenesis. Furthermore, this could illuminate new treatment options for GC with the emerging availability of novel biological therapies targeting the majority of cytokines. It is essential to better comprehend the part that these inflammatory mediators play in the process of gastric carcinogenesis.

To this end, our aim was to perform a tandem systematic review and meta-analysis, firstly investigating the cytokines induced by *H. pylori* infection and secondly assessing their association to GC. We observe that there was a distinct geographical difference in cytokine production associated with *H. pylori* infection. In addition, the level of Interleukin 6 (IL-6) induction in *H. pylori*-infected cases closely correlates to gastric cancer incidence in the corresponding global regions. Given that IL-6 is considered a promising therapeutic target in various malignancies and that it is druggable, our discovery may shed light on future mechanistic studies and clinical treatment of *H. pylori*-associated GC.

## 2 Materials and methods

### 2.1 Search strategy

Two separate systematic searches were performed: one to identify cytokines associated with *H. pylori* infection, and one to identify cytokines associated with GC. For the purpose of this systematic review and meta-analysis, we conducted a systematic search of all publicly available material found in Embase (1971-2022), Medline Ovid (1946-2022), Web of Science Core Collection (1975-2022), and the Cochrane Database (1992-2022). The following search terms were used: (cytokine OR chemokine OR interferon) AND (*Helicobacter pylori* OR *H. pylori*) AND (blood OR serum OR plasma OR biopsy) for the *H. pylori* meta-analysis, and (cytokine OR chemokine OR interferon) and (stomach cancer OR gastric cancer) and (blood OR serum OR plasma OR biopsy) for the GC meta-analysis. The full search strategies can be found in **Supplementary File 1**. The meta-analysis followed the PRISMA guidelines (16).

### 2.2 Study selection

Prior to the literature search, inclusion and exclusion criteria were specified. For studies to be included in this meta-analysis, they had to satisfy the following criteria: 1) written in English, Chinese or Dutch in peer-reviewed journals; 2) cytokine level assessment based on *ex vivo* sample measurement; 3) control group negative for *H. pylori*/free of stomach cancer described, 4) cytokine levels reported based on protein measurement. Studies were excluded if 1) they consisted of review articles, published abstracts, meta-analyses, editorials or case reports; 2) the method of *H. pylori* infection status determination was not described or gastric cancer status was not individually distinguished from other cancers; 3) cytokine levels were measured after *in vitro* stimulation of cells or in animal models; 4) quantitative cytokine levels were not reported for both cases and controls; 5) data was reported for ≤10 cases or controls.

### 2.3 Data extraction and study quality assessment

Two authors independently screened each article based on the title and abstract, to assess whether or not the research was based on clinical data, whether or not levels of cytokines were reported, whether or not the *H. pylori* infection was current, and whether or not the full text was accessible. Any disagreements were resolved through further discussion. Data were collected from selected studies from article text and tables. Studies without written description of cytokine levels were excluded (i.e. data was not extrapolated from figures). Main data extracted for the meta-analysis was sample size, mean cytokine concentrations, standard deviation and P-value. Additional data included were year of study, country or region of patient inclusion, study setting (e.g. alternative underlying diseases), method of analysis of inflammatory markers and cytokines levels, method of *H. pylori* detection. For all selected publications, bias was assessed using Newcastle–Ottawa scale, following the recommendation of the Musculoskeletal Group (17).

### 2.4 Statistical analysis

Mean and standard deviation (SD) for cytokines levels were used when reported. When median and interquartile range (IQR) were reported, SD was computed from these data as described by Wan et al. (18, 19), while means were derived using the method described by Luo et al. (18, 20), in order to maximize the number of studies eligible for inclusion. When different subgroups were presented, which for the purpose of this review should be viewed as one group, means and SD were combined as described by Altman et al. (21, 22). The META pack in R (version 4.2.0) was used for meta-analysis. Study heterogeneity was described using I^2^, which shows the proportion of variance attributable to factors between studies as opposed to sampling mistakes (23). We deemed I^2^ values of more than fifty percent to be indicative of substantial heterogeneity (24). If a study reported on several groups (e.g. various diseases), we compared *H. pylori*-positive and -negative groups that had the same disease status individually. We expected a significant heterogeneity since reported quantitative levels of inflammatory cytokines may be strongly influenced by patients’ status and different ELISA kits. A random effects model was used to construct a weighted mean difference and 95 percent confidence intervals (CI) for continuous outcomes data. In the calculation of the uncertainty (confidence interval) around results, this meta-analytic method incorporates both within-study and between-study variation. A random effects model, unlike a fixed effects model, assumes that the genuine effects change from one study to the next. Significant heterogeneity in the results of the included studies yields broader confidence ranges in random effects models as compared to fixed effect models (25). To describe quantitative differences in cytokine levels between *H. pylori*-positive and -negative cases in meta-analysis, we followed the suggestion from *Cochrane Handbook for Systematic Reviews of Interventions*, using the standardized mean difference (SMD) to a uniform scale. The SMD quantifies the magnitude of the intervention effect in each study in relation to the variability seen in that study. Consequently, regardless of the exact scales used to perform the measurements, studies where the difference in means is the same fraction of the standard deviation will have the same SMD (26).

We performed sub-analyses for the *H. pylori* meta-analysis by grouping the individual studies per global region, divided as Europe, Russia, Africa, America, Middle East, East Asia, and Southeast Asia (**Supplementary Table S1**). Only cytokines described in ≥10 studies were included in this analysis.

We analyzed the Pearson’s correlation between Age-Standardized Rate of Incidence of GC and the SMD of cytokines which our meta-analyses indicated were significant increased in both *H. pylori* infected individuals and GC patients. Data on the incidence of GC was retrieved from the International Agency for Research on Cancer, 2020 (Cancer today).

## 3 Results

### 3.1 Studies included for *H. pylori* meta-analysis

**Figure 1:**
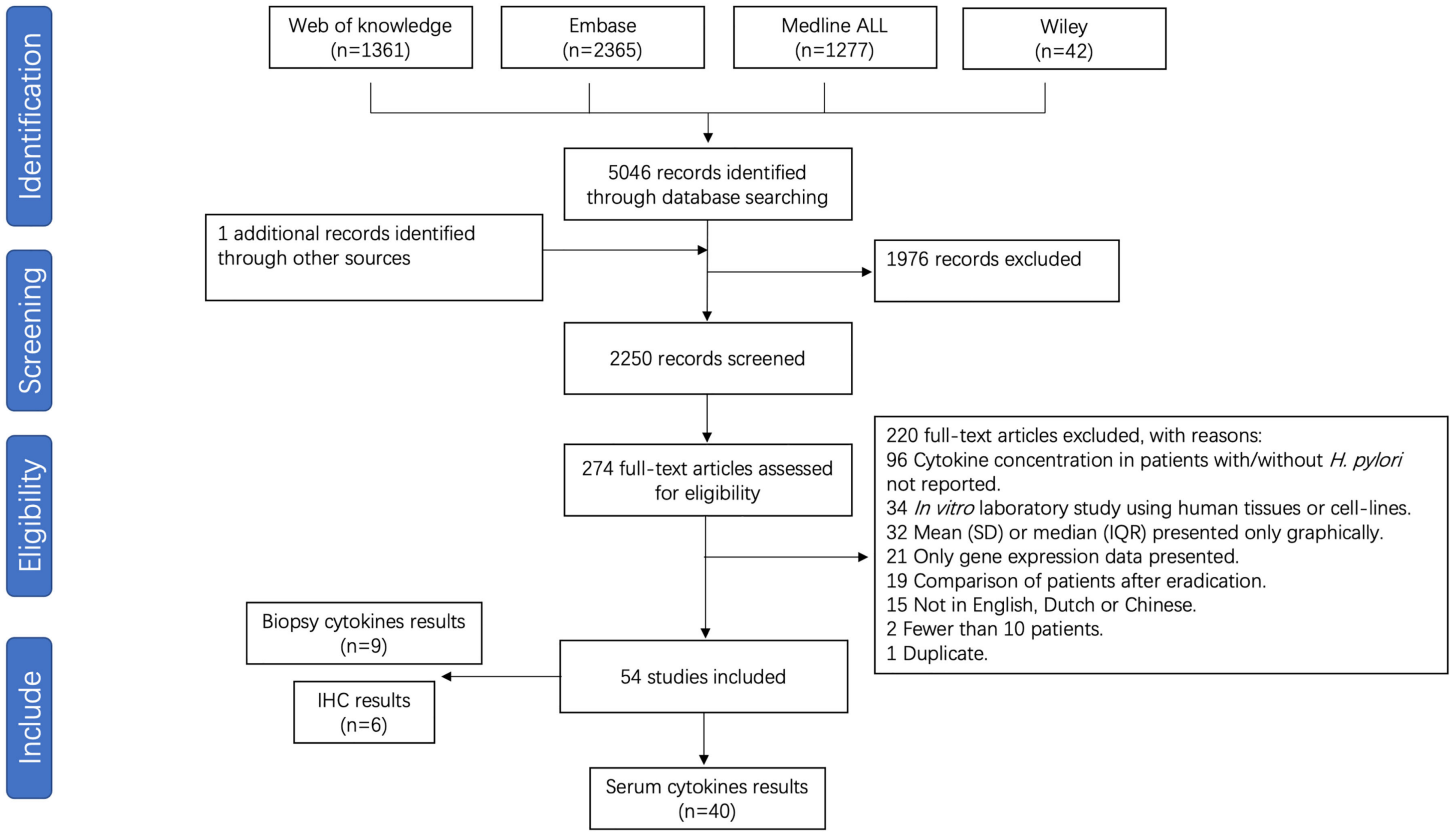
Flow chart of study selection for *H. pylori* infection meta-analysis.

After removal of duplicates, 2250 unique papers were identified, of which 274 full text manuscripts were assessed for eligibility. A total of 54 publications investigating the association between the pro-inflammatory cytokines and *H. pylori* infection were considered for inclusion in this systematic review, of which 40 studies were suitable for meta-analysis. A flow chart is presented in **Figure 1**. Included for analysis were 40 studies in which systemic cytokines were measured from serum. Nine studies measuring cytokines from biopsies through immune-based quantitative single or multiplex assays and 6 studies reporting on immunohistochemical analysis of formalin-fixed paraffin embedded (FFPE) sections, were not pooled in the analysis due to low numbers and different methods of reporting cytokine levels. Studies and patient data are summarized in **Supplementary Table S2**. The results of risk of bias analysis for each study are presented in **Supplementary Table S3**.

### 3.2 Serum cytokine alterations upon *H. pylori* infection

The following cytokines and chemokines were measured in relation to *H. pylori* infection in one or more studies: TNF-a, TNF-β, IFN-γ, CCL-1, CCL-2, CCL-3, CCL-7, CCL-8, CCL-11, CCL-13, CCL-15,CCL-17, CCL-19, CCL-20, CXCL-10, CXCL12, IL-1a, IL-1β, IL-2, IL-4, IL-5, IL-6, IL-7, IL-8, IL-10, IL-12, IL-13, IL-15, IL-16, IL-17A, IL-18,IL-27, IL-33, IL-35 and IL-37 (**Supplementary Table S4**). Only cytokines reported in ≥3 studies were pooled, leaving eight cytokines available for further analysis: IL-1β, IL-2, IL-4, IL-6, IL-8, IL-10, IFN-γ and TNFα (see **Table 1**).

**Table 1:** Standardized mean differences of cytokine levels in serum upon *H. pylori* infection.

		SMD results					Heterogeneity	
cytokine	Studies (n)	*H. pylori* positive cases (n)	*H. pylori* negative controls (n)	SMD	95%CI	p value	I^2^	p value
IL-1β	6	286	177	0.1	[-0.39; 0.59]	0.6815	79%	0.0002
IL-2	4	483	331	-3.99	[-8.86; 0.87]	0.1073	100%	< 0.0001
IL-4	5	257	195	1.84	[-0.63; 4.31]	0.1445	98%	< 0.0001
IL-6	22	2091	1620	1.93	[0.63; 3.23]	0.0036	98%	< 0.0001
IL-8	12	809	660	21.33	[-5.94; 48.60]	0.1253	99%	< 0.0001
IL-10	11	491	320	0.78	[-0.93; 2.48]	0.3731	96%	< 0.0001
IFN-γ	6	224	235	0.98	[0.02; 1.89]	0.0451	92%	< 0.0001
TNF-a	15	732	657	0.88	[0.48; 1.29]	< 0.0001	88%	< 0.0001

By far the most studied cytokine was IL-6, which was reported in 22 studies. In total, 3711 subjects were included in these studies. The overall SMD between *H. pylori*-infected and non-infected individuals was 1.93, 95%CI [0.63;3.23], indicating a large stimulatory effect of *H. pylori* infection on circulating IL-6 levels (P=0.0036). The second most commonly studied cytokine was TNF-α, which was investigated in 15 studies, including a total of 1224 individuals. On pooled analysis, serum TNF-α levels were found to be significantly increased upon *H. pylori* infection (SMD 0.88, 95%CI [0.46; 1.29], P ≤ 0.0001). In contrast, no effect of *H. pylori* infection on IL-8 levels was seen in 12 studies including 1469 subjects (SMD 21.33, 95%CI [-5.94; 48.60], P=0.1253). Similarly, no effect of *H. pylori* infection was observed in overall analysis of 11 studies investigating IL-10 levels in 811 subjects (SMD 0.78 95%CI [-0.93; 2.48], P=0.373). In addition, *H. pylori* infection did not modulate serum IL-1β (6 studies including 463 subjects, SMD 0.10, 95%CI [-0.39;0.59] P=0.682), IL-2 (4 studies including 452 subjects, SMD -3.99, 95%CI [-8.86; 0.87] P=0.1073), or IL-4 levels (5 studies investigating 452 subjects, SMD 1.84, 95%CI [-0.63;4.31] (P=0.144). IFN-γ, which was investigated in 459 individuals over 6 studies, did show significantly increased levels in serum upon infection with *H. pylori* (SMD 0.89, 95%CI [0.02;1.89] (P=0.0451).

### 3.3 Heterogeneity and sensitivity analysis

Significant heterogeneity was present in the SMD for the majority of the comparisons, justifying the use of a random effects model. This is consistent with other meta-analyses investigating cytokine levels (25, 27–29), and may be caused by unavoidable variation in, for example, different kit usage and different geographical locations. Another possible factor influencing heterogeneity in the *H. pylori* meta-analysis is the method by which *H. pylori*-infected individuals are identified, as some assays (e.g. pathology reports of *H. pylori* presence in gastric mucosa, urea breath test) signify current infection, while other methods (stool antigen, anti-*H. pylori* IgG levels) may also show positivity for some time after eradication of the bacterium. Thus, we performed a sensitivity analysis, removing 21 studies which used anti-*H. pylori* IgG levels to identity infection status. However, while significant differences for IL-6 and TNF-α serum cytokine levels upon *H. pylori* infection were still observed, heterogeneity was only marginally reduced (**Supplementary Table S5**). To further analyse heterogeneity, we investigated the impact of individual studies. Only two studies affected the I^2^ by more than 5%. Upon exclusion of these studies, IL-1β serum levels were no longer found to be significantly enhanced upon *H. pylori* infection (exclusion of Raman Mishra et al. (30) IL-1β SMD -0.06 [-0.49; 0.36], P=0.7678, exclusion of C. Roubaud-Baudron et al. (13) IL-1β SMD 0.27 [-0.15; 0.69], P=0.2039). None of the other cytokines were affected.

### 3.4 Sub-analysis of global differences in cytokine induction by *H. pylori*

**Figure 2:**
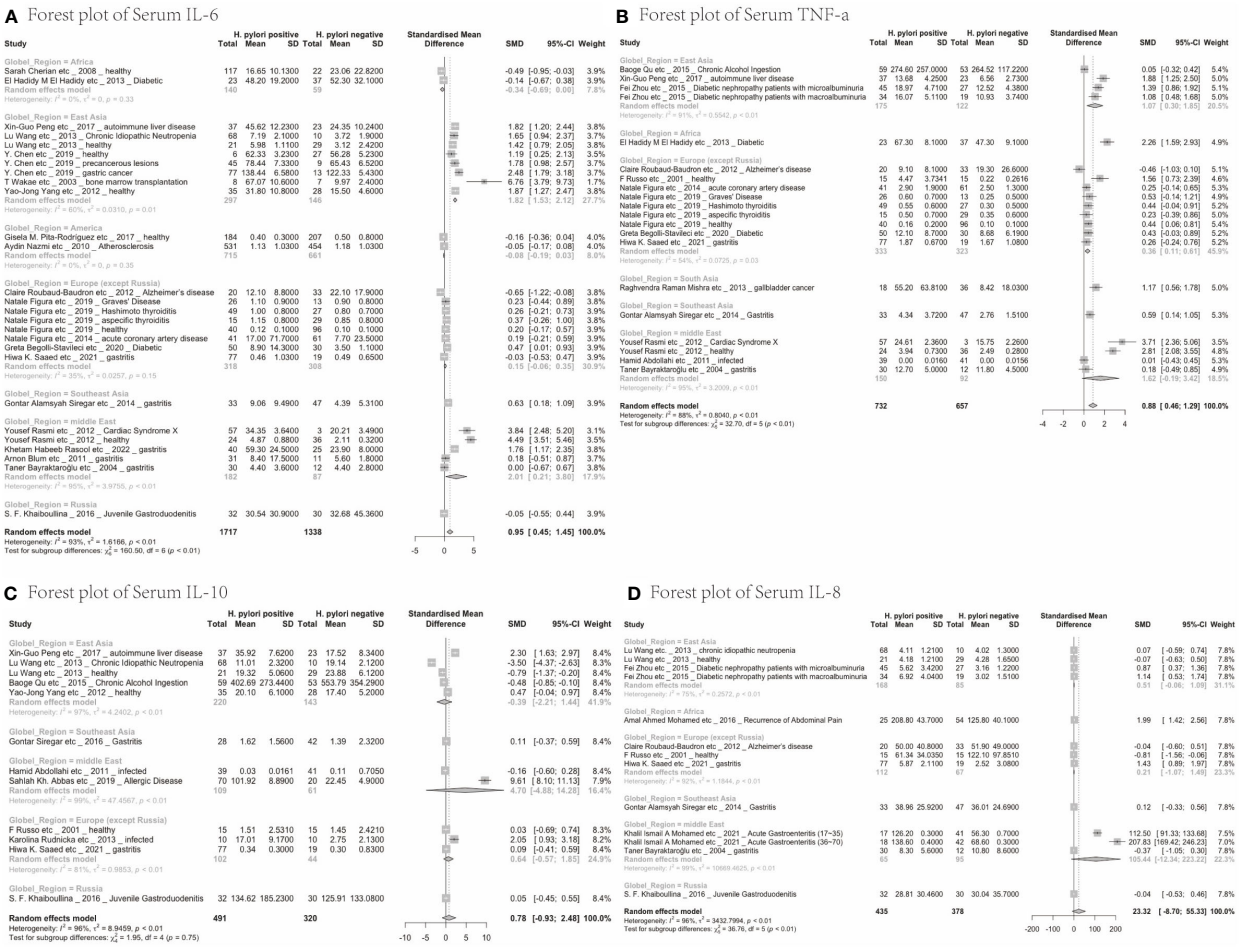
Forest plots of the meta-analysis of the effect of *H. pylori* infection on serum cytokines levels in different global regions. **(A)** IL-6 **(B)** TNF-a **(C)** IL-10 **(D)** IL-8.

Next, we analysed whether cytokine modulation upon *H. pylori* infection was dependent on the region of study. Only cytokines reported in ≥10 papers were included in this sub-analysis (**Table 2**). IL-6 levels were increased upon *H. pylori* infection in East Asia (4.60, 95%CI [1.48; 7.72]), the Middle East (2.01, 95%CI [0.21; 3.80]) and Southeast Asia (although based on only one study 0.63, 95%CI [0.18; 1.09]), but not North America, Europe, Russia and Africa, suggesting that regional differences exist for some inflammatory responses induced by *H. pylori* (visualized in **Figure 2A**). For TNF-α, sub-analysis of diverse global regions indicates that serum levels are significantly increased upon *H. pylori* infection in all global regions studied (East Asia: SMD 1.07, 95%CI [0.30; 1.85], Europe 0.36, 95%CI [0.11; 0.61], except the Middle East (**Figure 2B**). In contrast, IL-10 levels were not significantly affected in any individual region (**Figure 2C**), while sub-analysis of global regions indicates that IL-8 levels may be induced by *H. pylori* in Africa, although this only pertained to one study (SMD 1.99 95%CI [1.42;2.56], P ≤ 0.0001) (**Figure 2D**).

**Table 2:** Standardized mean differences of serum level of IL-6, IL-8, IL-10 and TNF-a upon *H. pylori* infection in different global regions.

		SMD results					Heterogeneity	
cytokine	Global Region	*H. pylori* positive cases (n)	*H. pylori* negative controls (n)	SMD	95%CI	p values	I^2^	p value
IL-6	East Asia	671	428	4.60	[1.48; 7.72]	0.0039	98.9%	<0.01
	Middle East	182	87	2.01	[0.21; 3.80]	0.0282	95%	<0.01
	America	715	661	-0.08	[-0.19; 0.03]	0.448	0%	0.35
	Europe (except Russia)	318	308	0.15	[-0.06; 0.35]	0.1637	35%	0.15
	Russia	32	30	-0.05	[-0.55; 0.44]	0.8294	NON	NON
	Africa	140	59	-0.34	[-0.69; 0.00]	0.0522	0%	0.33
	Southeast Asia	33	47	0.63	[0.18; 1.09]	0.0066	NON	NON
IL-8	East Asia	542	367	3.80	[-0.31; 7.91]	0.0700	99.4%	<0.01
	Middle East	65	95	105.44	[-12.34; 223.22]	0.0793	99%	<0.01
	Europe (except Russia)	112	67	0.21	[-1.07; 1.49]	0.7467	92%	<0.01
	Russia	32	30	-0.04	[-0.53; 0.46]	0.8852	NON	NON
	Africa	25	54	1.99	[1.42; 2.56]	< 0.0001	NON	NON
	Southeast Asia	33	47	0.12	[-0.33; 0.56]	0.6101	NON	NON
IL-10	East Asia	220	143	-0.39	[-2.21; 1.44]	0.6768	97%	<0.01
	Middle East	109	61	4.7	[-4.88; 14.28]	0.3364	99%	<0.01
	Europe (except Russia)	102	44	0.64	[-0.57; 1.85]	0.3018	81%	0.01
	Russia	32	30	0.05	[-0.45; 0.55]	0.8347	NON	NON
	Southeast Asia	28	42	0.11	[-0.37; 0.59]	0.6499	NON	NON
TNF-a	East Asia	175	122	1.07	[0.30; 1.85]	0.0067	91%	0.01
	Middle East	150	92	1.62	[-0.19; 3.42]	0.0787	95%	0.01
	Europe	333	323	0.36	[0.11; 0.61]	0.005	54%	0.03
	Africa	23	37	2.26	[1.59; 2.93]	< 0.0001	NON	NON
	Southeast Asia	33	47	0.59	[0.14; 1.05]	0.011	NON	NON
	South Asia	18	36	1.17	[0.56; 1.78]	0.0002	NON	NON

### 3.5 Studies included for gastric cancer meta-analysis

**Figure 3:**
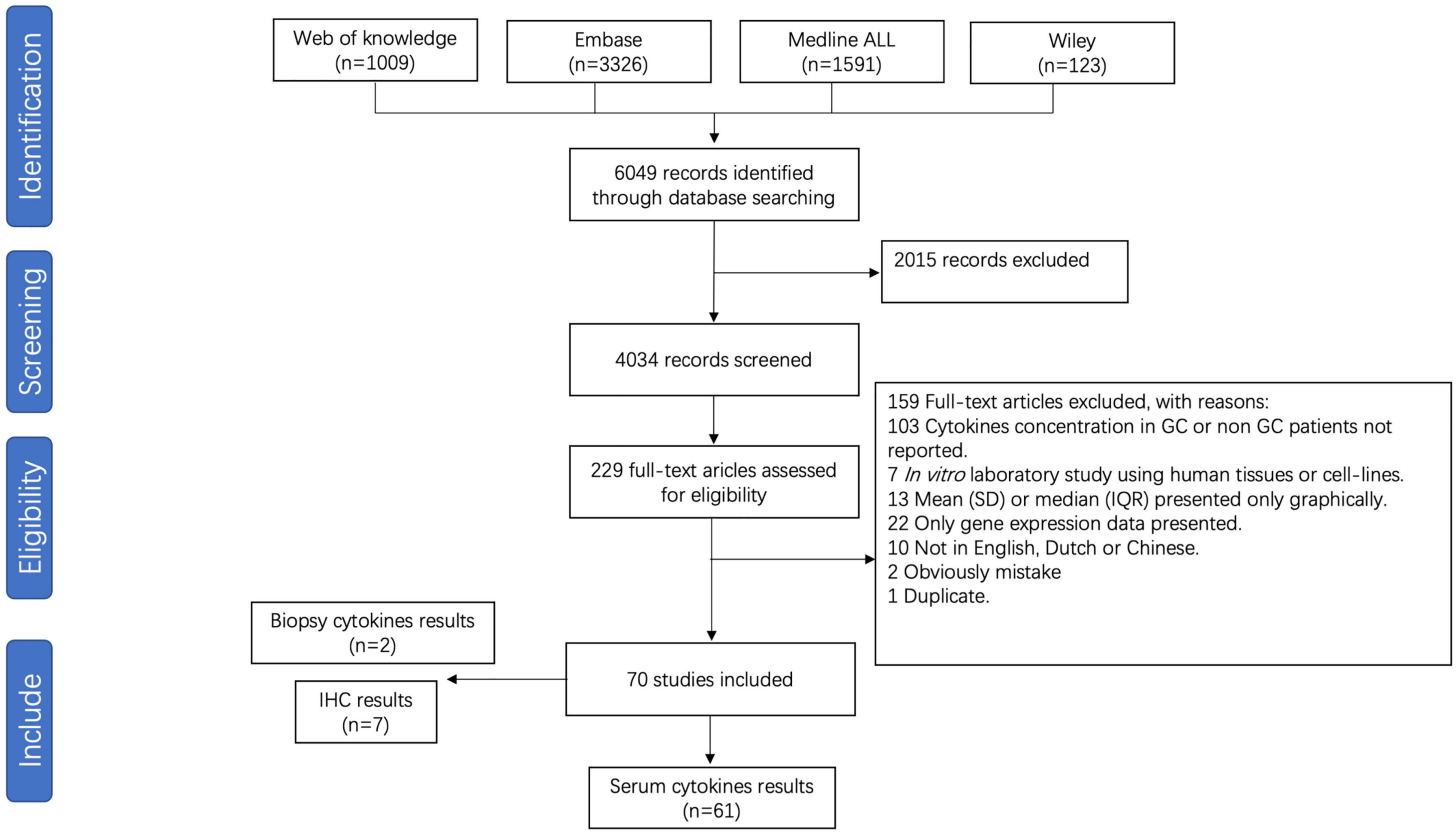
Flow chart of study selection for gastric cancer meta-analysis.

For meta-analysis of cytokine levels associated with GC, 4034 abstracts were screened, resulting in 229 full length manuscripts considered for eligibility. A total of 70 publications investigated the association between pro-inflammatory cytokines and GC, of which 61 studies investigating cytokine serum levels were included for meta-analysis (**Figure 3**). The details of all included studies are presented in **Supplementary Table S6**. Nine studies investigating cytokine levels in gastric biopsies through either immune-based quantitative analysis or immunohistochemistry were excluded due to low numbers. The risk of bias is presented in **Supplementary Table S7**.

### 3.6 Serum level of cytokines in gastric cancer patients

The 61 studies included compared TNF-α, TNF-β, IFN-r, CCL-2, CCL-5, CCL-18, CCL-20, CXCL-8, CXCL11, CXCL12, CXCL13, IL-1a, IL-1β, IL-2, IL-4, IL-5, IL-6, IL-7, IL-8, IL-10, IL-11, IL-12, IL-13, IL-15, IL-16, IL-17, IL-18, IL-23, IL-24, IL-26, IL-29, IL-32, IL-35, IL-37, TGF-β, TGF-β1, TGF-β2, IFN-λ1 and IFN-λ2/3 serum cytokine levels between GC patients and controls (**Supplementary Table S8**). Cytokines investigated in three or more studies were IL-1β (7 studies; 1308 individuals included), IL-2 (5 studies, 1127 individuals), IL-4 (6 studies, 1148 subjects), IL-6 (19 studies; 2804 subjects), IL-7 (3 studies; 466 individuals included), IL-8 (12 studies; 2455 individuals), IL-10 (13 studies, 2512 individuals), IL-12 (7 studies; 1016 individuals), IL-17 (7 studies; 783 individuals), IL-18 (3 studies; 234 individuals), IL-33 (3 studies; 316 individuals), TNF-α (12 studies, 1642 individuals), TNF-β (3 studies, 506 individuals), and IFN-γ (12 studies, 1896 individuals) (**Table 3**).

**Table 3:** Standardized mean difference of serum cytokine levels between gastric cancer cases and controls.

		SMD					heterogeneity	
cytokine	studies (n)	gastric cancer patients (n)	controls (n)	SMD	95%CI	P	I2	p value
IL-1β	7	564	744	0.76	[-0.73;2.25]	0.3194	97%	<0.01
IL-2	5	520	607	2.89	[-2.20;7.97]	0.2663	99%	<0.01
IL-4	6	494	654	0.95	[0.00;1.91]	0.0503	97%	<0.01
IL-6	19	1590	1214	1.64	[0.88;2.39]	<0.0001	97%	<0.01
IL-7	3	288	178	1.04	[0.45;1.63]	0.0006	89%	<0.01
IL-8	12	1115	1340	2.08	[-0.17;4.33]	0.0695	98%	<0.01
IL-10	13	1221	1291	2.17	[0.63;3.70]	0.0056	98%	<0.01
IL-12	7	605	411	2.1	[0.91;3.29]	0.0005	96%	<0.01
IL-17	7	456	327	0.88	[-0.11;1.87]	0.0823	98%	<0.01
IL-18	3	158	76	0.53	[-0.08;1.15]	0.0896	80%	<0.01
IL-33	3	144	172	2.11	[-2.23;6.44]	0.3405	99%	<0.01
TNF-α	12	755	887	2.34	[0.29;4.38]	0.0251	99%	<0.01
IFN-γ	12	951	945	0.44	[-1.01;1.89]	0.5532	98%	<0.01

A significant upregulation of IL-6 serum levels was seen in GC patients (SMD: 1.64, 95%CI [0.88; 2.39], P<0.0001). Other cytokines showing a significant increase in serum from GC patients compared to controls were IL-7 (SMD: 1.04, 95% CI [0.45; 1.63], P=0.0006), IL-10 (SMD: 2.17, 95%CI [0.63; 3.70], P=0.0056), IL-12 (SMD: 2.1, 95%CI [0.91; 3.29], P=0.0056), and TNF-α (SMD: 2.34, 95%CI [0.29; 4.38], P=0.0251). While heterogeneity was high for all cytokines, none of the studies individually affected the I^2^ with more than 5%.

### 3.7 Correlation between *H. pylori*-induced cytokine levels and global gastric cancer incidence

**Figure 4:**
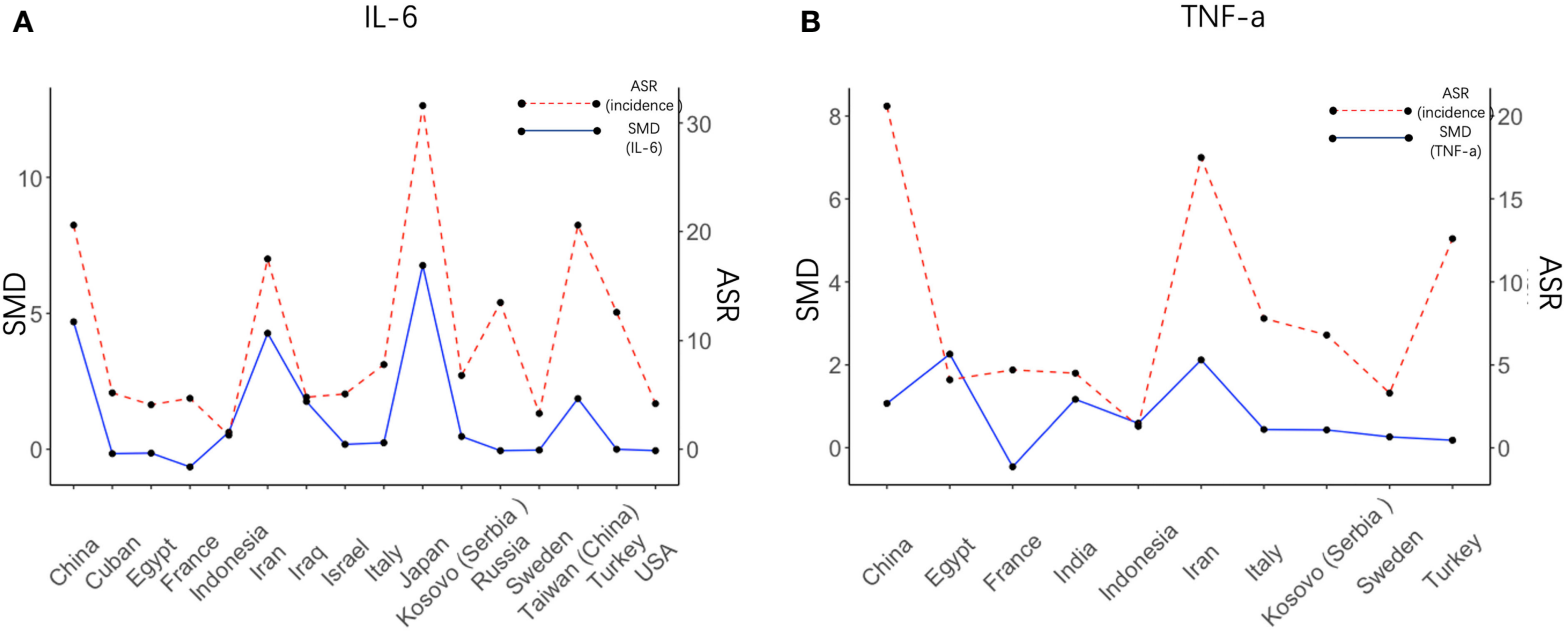
Regional IL6 levels induced by *H. pylori* correlate with cancer risk in these regions. Visual representation of the correlation between the SMD of IL-6 **(A)** and TNF-α **(B)** after *H. pylori* infection in different countries (left axis) and the value of the risk of gastric cancer in these countries (right axis). SMD, Standardized Mean Difference; ASR, Age-Standardized Rate (incidence).

We next set out to explore the relationship between serum cytokine changes upon *H. pylori* infection and the risk of GC development. IL-6 and TNF-α were the only cytokines presenting a significant increase upon both *H. pylori* infection and gastric cancer development. Therefore, serum levels of IL-6 and TNF-α after *H. pylori* infection were calculated individually for each country/region (**Figure 4**) and correlated to the GC incidence in this same region. SMD of IL-6 levels presented a significant correlation with the relative incidence of GC (R = 0.85, p= 0.000035, see also **Supplementary Figure S1A**). Visualization of the relationship between IL-6 levels (SMD) and the risk of GC using a bubble plot shows that in countries and regions with an age standardized rate (ASR) greater than the global average, higher IL-6 levels are induced upon *H. pylori* infection than in countries with a relative GC risk below the global average (**Supplementary Figure S1C**). However, such a correlation was not observed for TNF-α (R = 0.29, p= 0.41, **Figure 4B**; **Supplementary Figures S1B, S1D**).

## 4 Discussion

*H. pylori*-induced inflammation is commonly regarded as a crucial factor in the development of GC. However, there are currently no biomarkers that can accurately predict the risk of non-inherited gastric cancer (31). In this study, we aimed to perform a systematic review and tandem meta-analysis in order to investigate whether *H. pylori* infection is associated with an upregulation of specific inflammatory cytokines related to GC development. Our results indicate that IL-6 and TNFα are the main cytokines induced upon *H. pylori* infection, and are also increased in GC patients. Levels of IL-6 correlate with GC risk in different countries, strongly supporting a role for this cytokine in the development of this lethal disease. GC incidence varies widely worldwide, with the highest incidence observed in East Asia, and the lowest incidence rates in Africa (32). Regional differences in GC incidence may be caused by differences in diet, hygiene and genetic factors. However, one of the main explanations may be the substantial geographic heterogeneity of *H. pylori* strains. Different *H. pylori* strains carry different combinations of genes related to their virulence and pathogenicity. CagA is one of the most extensively investigated of these pathogenicity factors and has been categorized as an oncogenic protein (33). While CagA is present in just 60-70% of Western isolates, nearly all strains in East Asia carry CagA (34). In addition, gastritis patients infected with East Asian CagA-positive strains present with much higher gastric inflammation, gastritis and atrophy as compared to patients infected with CagA-negative or Western CagA-positive strains (35). *In vitro* experimentation has shown different cellular effects including cytokine induction by *H. pylori* strains with divergent virulence factors, which may account for discrepancies in regional cytokine pattern changes upon *H. pylori* infection as well as local differences in GC development. Thus, it is essential to take the region of study into consideration when studying *H. pylori*-induced serum cytokine levels.

Despite obtaining the highest pooled SMD, our results indicate that IL-8, which has been widely considered as one of the most critical cytokines modulated by *H. pylori*, is not globally increased in serum from *H. pylori*-infected individuals. While IL-8 was shown to be the single most up-regulated gene in gastric epithelial cells exposed to *H. pylori* (36), serum presents the accumulated release of cytokines from epithelial, immune and other cells. Similarly, IL-1β and IL-10 have been regarded as important cytokines mediating *H. pylori* effects, but while some studies showed a significant up-regulation, the pooled SMD did not present a significant increase in this meta-analysis.

In contrast, results from our study add to the evidence that IL-6, IFN-γ, and TNF-α are raised after *H. pylori* infection. It is particularly notable that the increase in IL-6 was most substantial in East Asia and the Middle East, which are the specific regions with the highest rate of stomach cancer (37).

GC can arise years after initial *H. pylori* infection, and it is as yet unclear to what extent persisting cytokine presence may contribute to this phenomenon. We found that levels of IL-6, IL-7, IL-10, IL-12, and TNF-α were significantly increased in GC patients, of which only IL-6 and TNF-α were also raised upon *H. pylori* infection in our analysis. Cancer poses its own inflammatory state, which may account for upregulated cytokines that are not induced upon initial *H. pylori* infection. While *H. pylori*-mediated TNFα induction did not show a correlation with GC incidence, IL-6 levels did, suggesting that perhaps initial induction of TNFα is not a direct driver of carcinogenesis. Alternatively, the fewer studies reporting on TNFα levels may have resulted in an underestimation of its effect.

The present study revealed that IL-6 is one of the most critical inflammatory cytokines in the *H. pylori* associated-gastric carcinogenesis. Mechanistically, IL-6 stimulates gastric inflammation, and it stimulates the proliferative response of gastric cells in order to restore stomach function (38). However, repeated gastric injury leads to the development of chronic gastritis and inflammation-induced stomach cancer. IL-6 has can have several cancer-stimulatory effects, many of which are driven by activation of the janus kinase (JAK)-signal transducer and activator or transcription (STAT)3 signaling pathway, which in turn triggers the transcription of a sequence of genes that are responsible for cell proliferation, apoptosis inhibition, cell cycle progression and modulation of the extracellular matrix (39, 40).

We noted a significant degree of heterogeneity in our analysis, which may be attributable to differences in cytokine ELISA kits. In addition, some studies have demonstrated complex and variable alterations of cytokine levels after *H. pylori* infection related to factors such as age of patients, the location of the infection, the histological subtype of stomach cancer, and the stage of the disease (41–47). However, we were unable to add these variables to our analyses as detailed information was not available for most of these factors. Moreover, we only included data from 27 countries, based on all available studies. While these represent a diverse range of regions and most of the population in the world, the results may not be fully generalizable to other countries. In addition, it should be noted that many studies reported *H. pylori*-induced cytokine levels in the context of another underlying disease, and while we only included those studies that presented disease-specific controls, it is conceivable that the *H. pylori*-induced changes were masked due to the underlying illness. Lastly, our primary conclusion is based on country-level summaries. However, with the migration of various demographic groups, individual studies may have included local populations of a different demographic background as the GC incidence which is reported for a whole country. However, while IL-8 and TNFα did not show regional expression differences correlating with GC incidence, IL-6 levels were strongly correlated with GC risk, suggesting that our results can reasonably reflect the link between cytokines and the incidence of GC in diverse countries.

## 5 Conclusion

In conclusion, this study demonstrates that IL-6 and TNF-α levels are upregulated in both *H. pylori*-infection and GC. In particular IL-6 shows location-specific increases which correlate to GC incidence in these regions, identifying this cytokine an important causative candidate. Further investigation is warranted to characterize this correlation and verify the role of IL-6 in driving GC development.

## Data availability statement

The original contributions presented in the study are included in the article/**Supplementary Material**. Further inquiries can be directed to the corresponding author.

## Author contributions

BY: Study design, acquisition, analysis and interpretation of data, manuscript drafting. GF: Acquisition of data, manuscript revising, final approval of the manuscript. LX: Acquisition, analysis and interpretation of data, critical appraisal of manuscript. MP: Critical appraisal and final approval of the manuscript. All authors contributed to the article and approved the submitted version.
