# Supplementary material for: Overlapping cytokines in H. pylori infection and gastric cancer: A tandem meta-analysis
Source: Front Immunol. 2023 Mar 15;14:1125658. doi: 10.3389/fimmu.2023.1125658 (PMC10050690; doi:10.3389/fimmu.2023.1125658)
Supplement: Supplementary file 1 [file DataSheet_1.pdf]

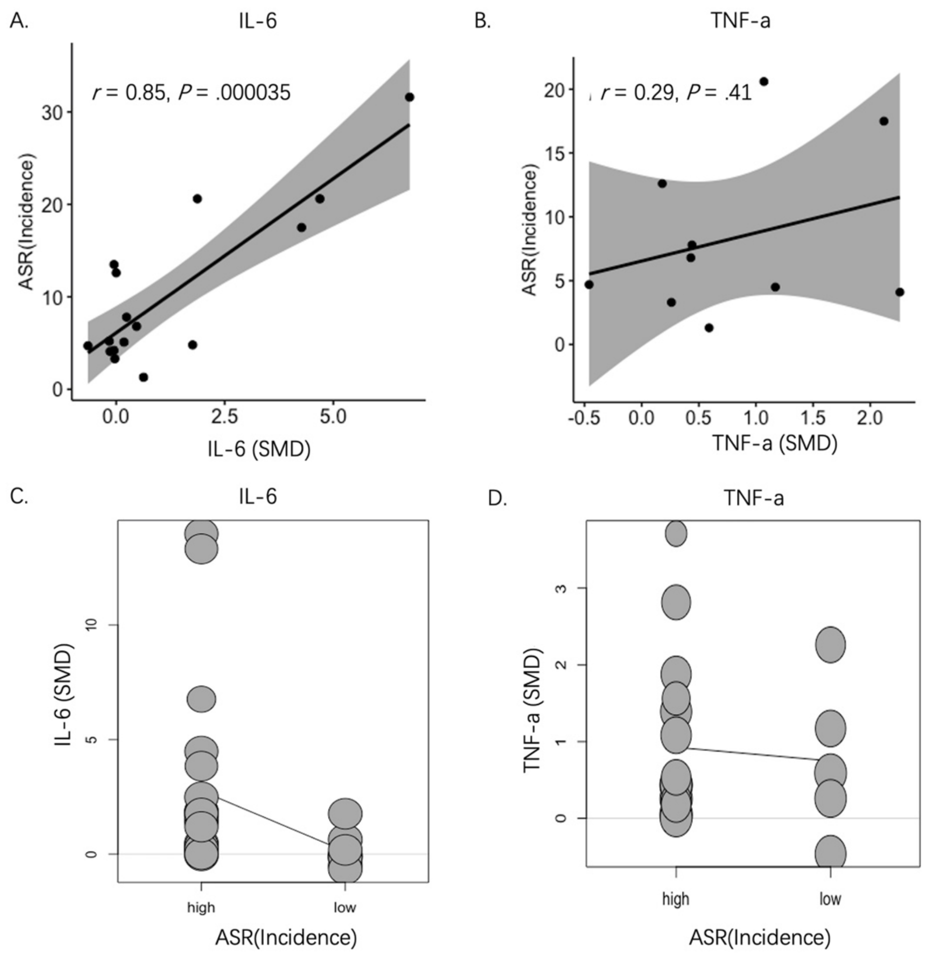

Figure S1. (A) Pearson correlation between SMD of serum IL-6 levels and age standardized risk of incidence. (B) Pearson correlation between SMD of serum TNF-α levels and age standardized risk of incidence. (C) bubble plot of relationship between GC risk (incidence) and SMD of serum IL-6 level. (D) bubble plot of relationship between GC risk (incidence) and SMD of serum TNF-α level.

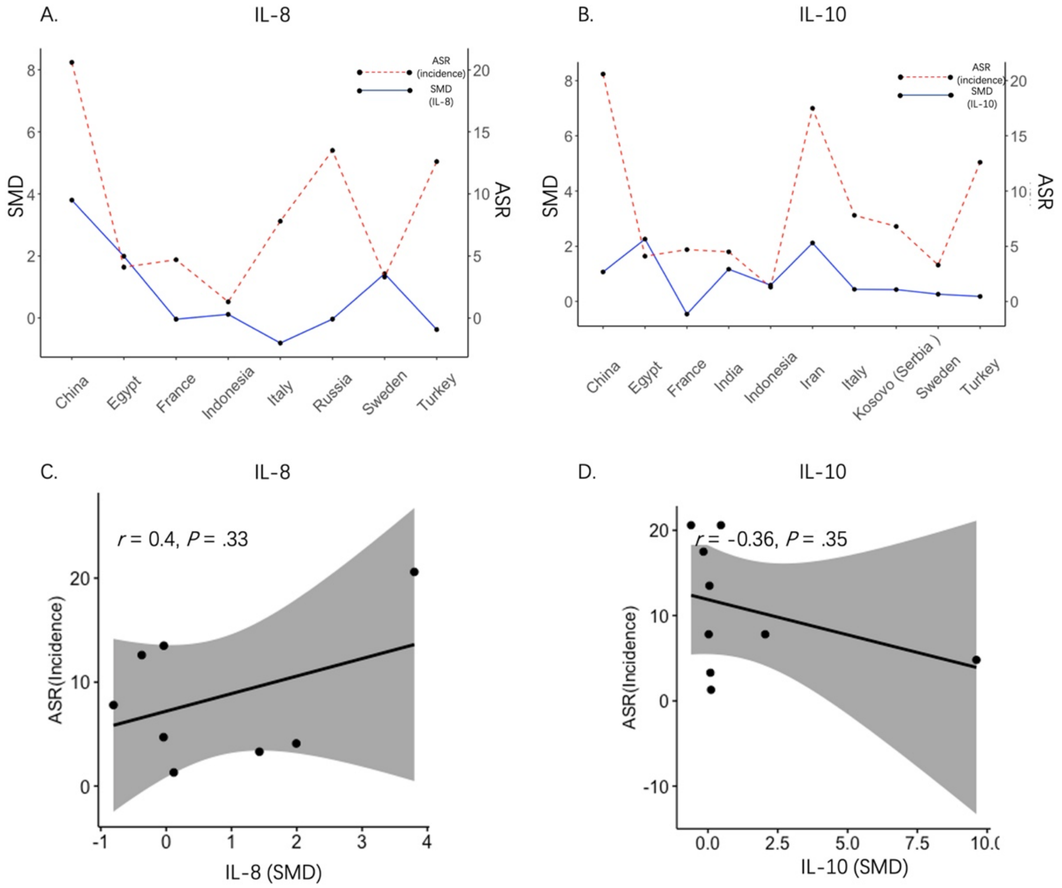

Figure S2. Visual representation of the correlation between the SMD of IL-8 (A) and IL-10 (B) after H. pylori infection in different countries (left axis) and the value of the risk of gastric cancer in these countries (right axis). SMD: Standardized Mean Difference, ASR: Age-Standardized Rate (incidence). (C) Pearson correlation between SMD of serum IL-8 levels and age standardized risk of incidence. (D) Pearson correlation between SMD of serum IL-10 levels and age standardized risk of incidence.
